# Leiomyomatosis peritonealis disseminata with omental involvement: a case report

**DOI:** 10.3389/fgwh.2026.1754975

**Published:** 2026-05-07

**Authors:** Lin Qin, Wenjie Shen

**Affiliations:** Senior Department of Obstetrics & Gynecology, the Seventh Medical Center of PLA General Hospital. Beijing, China

**Keywords:** fibroid morcellator, laparoscopy, leiomyomatosis peritonealis disseminata (LPD), omental involvement, uterine leiomyoma

## Abstract

Uterine leiomyoma is a benign tumor of the uterus, and laparoscopic myomectomy is a common procedure for young women. We conducted a retrospective analysis of one patient admitted to our hospital. After undergoing two laparoscopic myomectomies at other hospital, surgical records from the referring hospital indicated that a containment bag was used. However, a pelvic mass reappeared, which was confirmed during surgery to be leiomyomatosis peritonealis disseminata with omental involvement. This indicated that even with a containment bag, dissemination still occurred. We also reviewed relevant literature to better understand the risks associated with laparoscopic myomectomy. The exact mechanism of dissemination cannot be definitively determined from this single case alone. Additionally, close follow-up after surgery is essential to prevent recurrence and malignant transformation.

## 1 Introduction

Uterine leiomyoma is a common benign tumor in women (1). The incidence rate among women aged 40 to 50 years is as high as 51.2%∼60% (2, 3). This article retrospectively analyzed a case involving two laparoscopic myomectomies performed at other hospital, and discussed the diagnosis and treatment of leiomyomatosis peritonealis disseminata with omental involvement. It also examined the impact of laparoscopic myomectomy on treatment outcomes and survival prognosis in such patients.

## 2 Case report

The patient was a 46-year-old female admitted to our hospital on March 12, 2024, following the incidental discovery of a pelvic mass 10 months prior. The patient denied any history of abdominal pain or bloating, reports no anemia, and had experienced no recent weight changes. Menstrual history: regular menstrual cycles lasting 5 days, occurring every 30 days, with moderate flow and no dysmenorrhea. BMI was 23. Obstetric history: G1P1. The patient had no history of hormone exposure. She denied abdominal pain, pressure symptoms, weight changes, or anemia. The lesion was asymptomatic and found incidentally. In 2015, she underwent laparoscopic myomectomy for uterine leiomyoma. In 2022, a second laparoscopic myomectomy was performed, and both surgical reported noted the use of a sealed retrieval bag for rotational resection of the leiomyoma. There was no regular follow-up after either surgery. In May 2023, ultrasound revealed multiple intramural myomas within the uterine muscle walls, the largest measuring 2.8 cm × 2.2 cm. Additionally, a hypoechoic mass measuring 8.9cm × 4.8 cm was observed anterior to the uterus, with clear boundaries. Differential diagnoses included broad ligament myoma or uterine sarcoma. The patient was admitted for further diagnosis and treatment. Pelvic examination revealed normal external genitalia, an unobstructed vagina, and a smooth cervix. The uterus was anteverted, irregular in shape, enlarged to the size consistent with over 3 months of pregnancy, non-tender, mobile, and no obvious abnormalities were detected in the bilateral adnexa.

**Figure 1:**
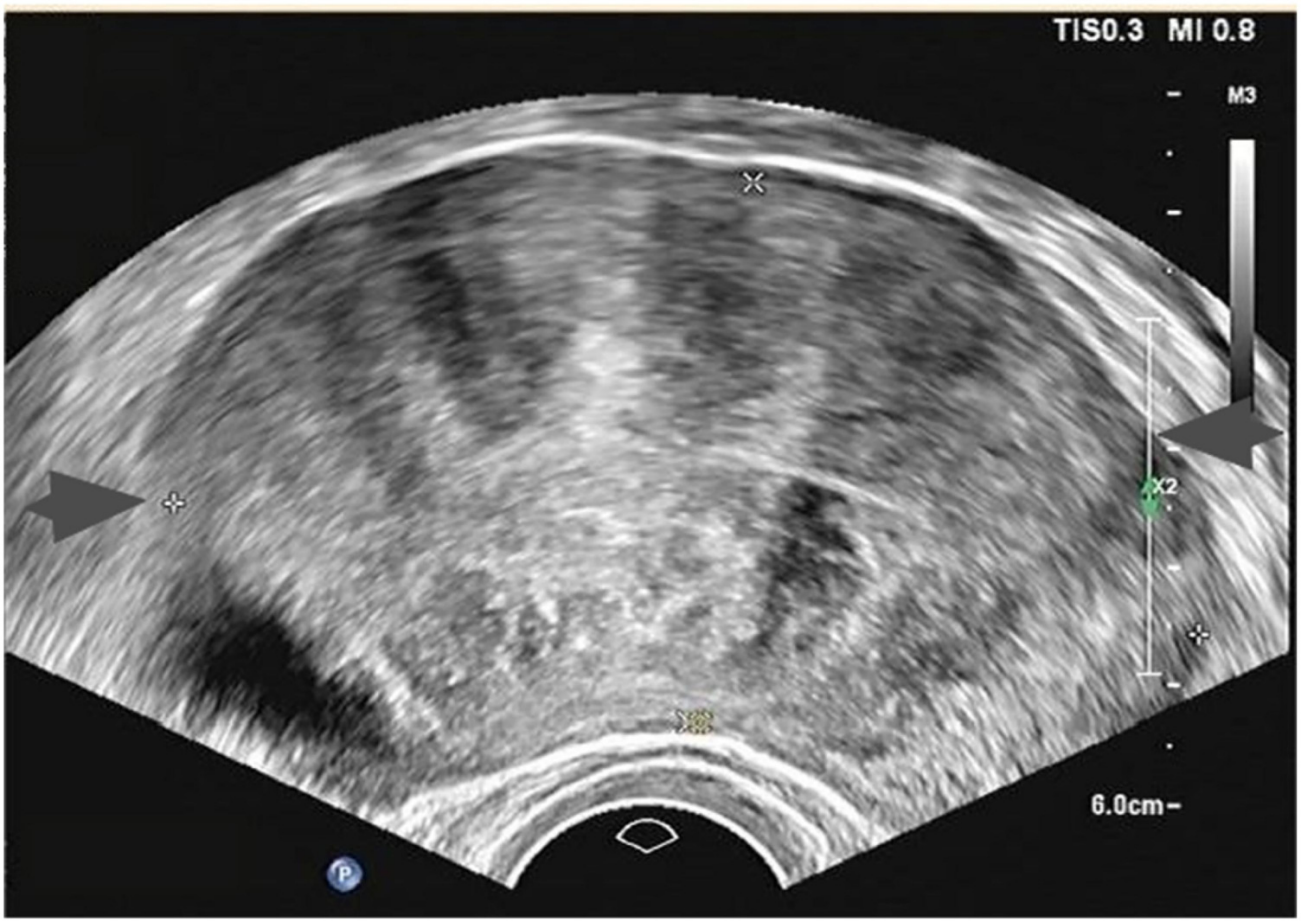
Ultrasound showed pelvic mass.

After admission, relevant examinations were completed. There were no abnormalities detected in ThinPrep Cytologic Test (TCT) and Human Papillomavirus (HPV) screening, and tumor markers were within normal limits (CA125: 21.20 U/mL; CA19-9: 5.18 U/mL). Ultrasonography revealed a normal-sized uterus, with heterogeneous echoes in the myometrium. Multiple hypoechoic nodules were observed within the myometrium, with the largest located beneath the serosa, measuring approximately 8.6 cm × 4.6 cm, exhibiting clear boundaries and heterogeneous internal echoes. Color Doppler Flow Imaging (CDFI) demonstrated limited blood flow signals within these nodules, consistent with the ultrasound characteristics of multiple myomas (the largest located subserosally), as shown in Figure 1. Routine blood test, urinalysis, hepatic and renal function tests, and coagulation profiles were all within normal ranges. Based on the patient's condition, uterine sarcoma was not be excluded. The patient had no desire for future fertility and did not wish to the uterus. For patients with a history of fibroids, a significant short-term increase fibroid size, it was highly suggestive of sarcoma. MRI could be valuable in differentiating between benign and malignant uterine tumors. The patient underwent contrast-enhanced MRI at an external hospital; however, the report did not indicate the presence of sarcoma. Uterine fibroids and uterine sarcomas were difficult to distinguish through preoperative imaging and auxiliary examinations. Because uterine sarcoma must be excluded before surgery, a rapid frozen section examination was planned during the operation. If the frozen section indicates uterine sarcoma, the surgical scope will need to be expanded. Parasitic leiomyoma refers to the spontaneous or iatrogenic detachment of uterine fibroids from the uterine body, which then develop new blood vessels and implant into other tissues or organs, such as the peritoneum, intestinal tract, or other sites. It typically presents as an isolated large solid nodule. There is overlap between parasitic leiomyoma and LPD, especially in secondary cases following laparoscopic surgery, and currently, no clear distinction exists. Gastrointestinal Stromal Tumors (GIST) often present with nonspecific symptoms; and as the lesion grows, it may cause abdominal pain, a palpable mass, bleeding, or obstruction. GIST can develop anywhere along the gastrointestinal tract, including the omentum, mesentery, and peritoneum, but they most commonly occur in the stomach and small intestine. Some patients may present with acute abdominal conditions such as tumor rupture, perforation, or obstruction. Approximately 20% of GISTs are asymptomatic and are discovered incidentally. Definitive diagnosis is typically established through postoperative pathological examination. Although the patient did not report any symptoms such as gastrointestinal pain, the possibility of GIST cannot be excluded. Prior to surgery, a general surgeon has been consulted, and their assistance may be required during the procedure. The purpose of this surgery has been explored, and the surgical approach will be determined based on intraoperative findings.

**Figure 2:**
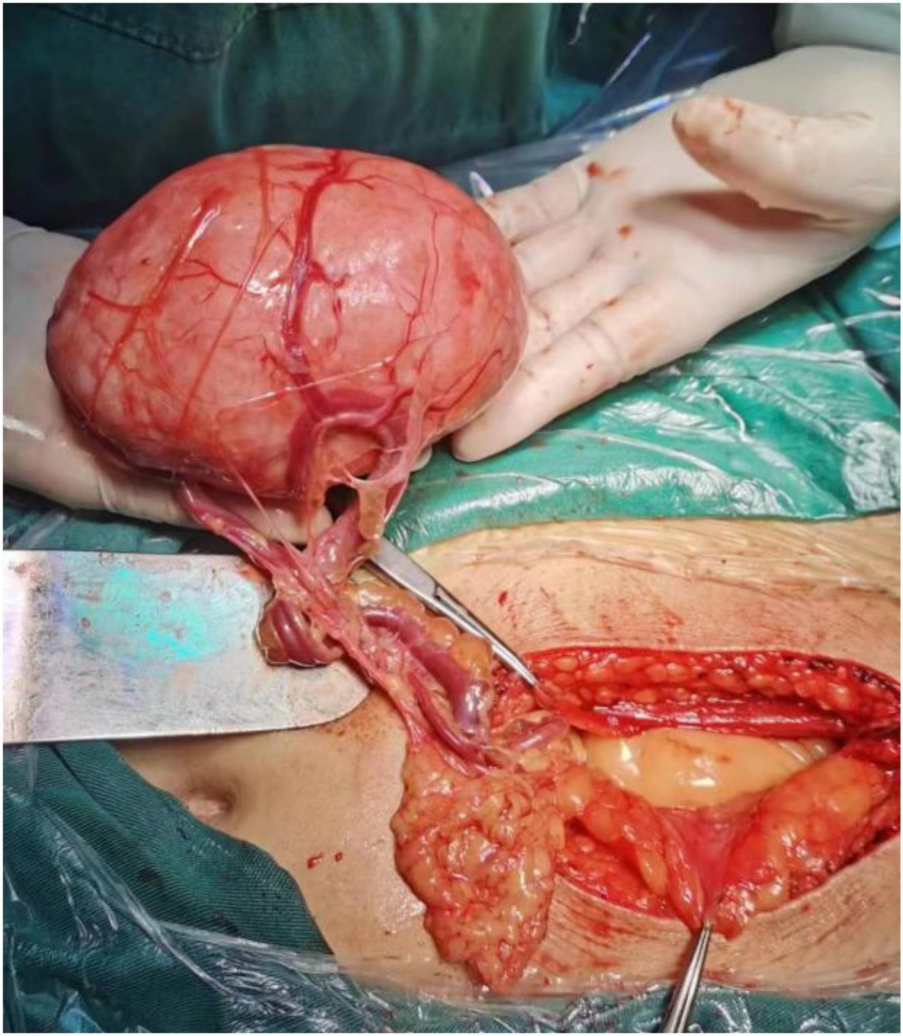
Intraoperative LPD with omental involvement.

**Figure 3:**
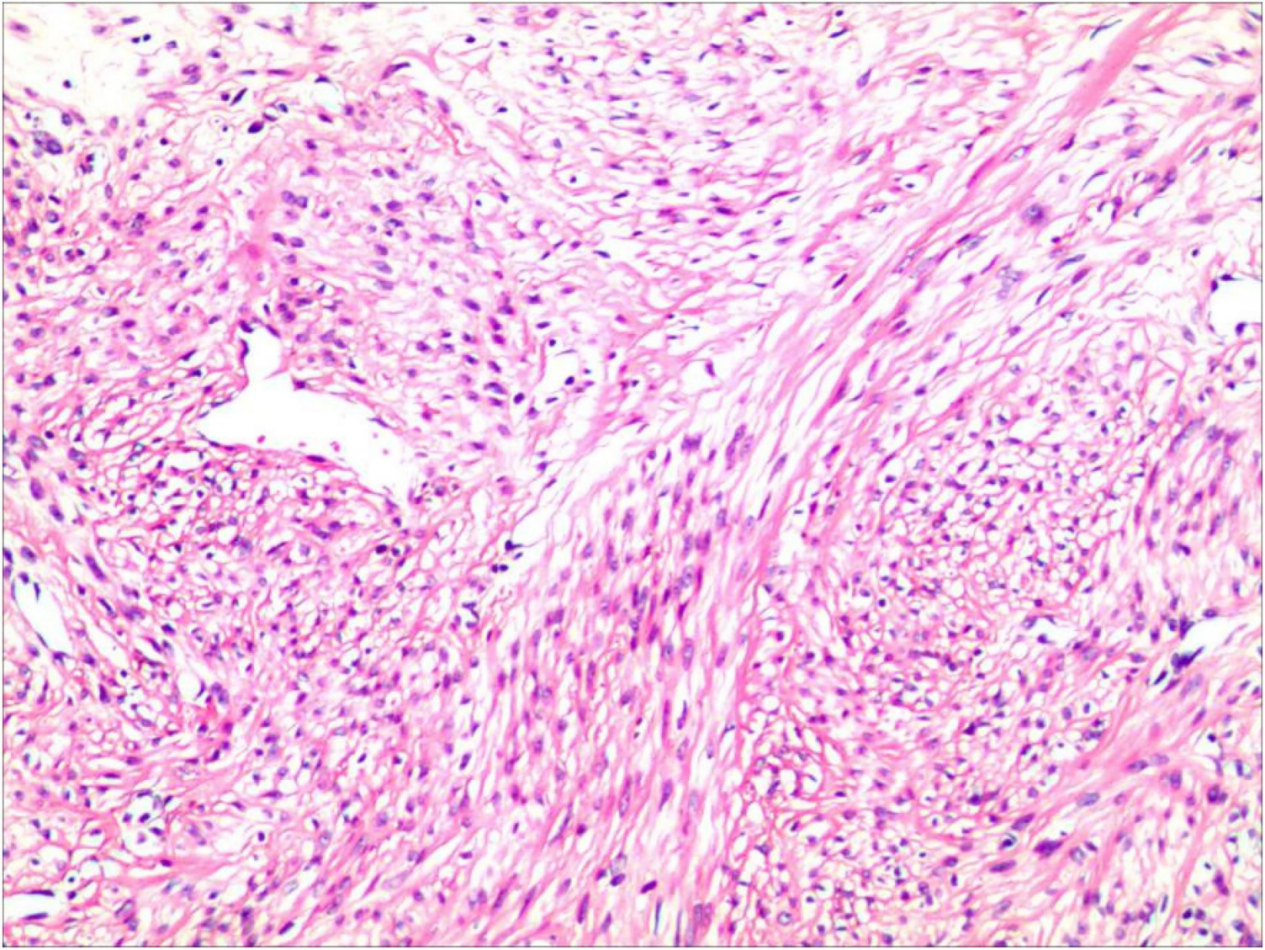
Postoperative biopsy (HE × 200).

On March 21, 2024, under general anesthesia, an exploratory laparotomy was performed, followed by a hysterectomy and omental myomectomy. Intraoperative findings revealed: there was 1 myoma measuring 12 cm on the surface of the omentum, and two fibroid nodules, each 1 cm in diameter on the peritoneum, as shown in Figure 2. The uterus was of normal size, with multiple myomas on the surface, each measuring approximately 1 to 2 cm in diameter. The fundus of the uterus was adhered to the intestinal membrane, while the appearance of both fallopian tubes and ovaries was normal. Intraoperative frozen section confirmed the diagnosis of myoma. Postoperative pathology also confirmed myoma, with a size of 11.0 cm × 8.0 cm × 6.5 cm, as shown in Figure 3. The tumor nodule consisted of proliferating spindle cells arranged in bundles. The cells had abundant eosinophilic cytoplasm, and rod-shaped nuclei. No necrosis or mitotic figures were observed. IHC: SMA(+), Desmin(+), CD10(-). Postoperative diagnosis: Leiomyomatosis Peritonealis Disseminata (LPD) with omental involvement. The patient recovered well after surgery and was scheduled for regular outpatient follow-up. Follow up until January 2026 showed good recovery with no signs of recurrence on B-ultrasound imaging.

## 3 Discussion

Uterine myoma is the most common benign tumors of the female reproductive organs (4) and one of the most prevalent tumors in the human body. Pelvic and extrauterine leiomyomas refer to leiomyomas that occur outside the uterus, in other organs and spaces within the pelvic cavity (5). The development of this type of myoma is related to estrogen stimulation (6) and shares the same pathological characteristics as uterine myomas. However, these myomas are relatively rare, and most exhibit varying degrees of degeneration, necrosis, cystic changes, and other alterations. Their clinical symptoms and imaging findings are nonspecific, resulting in a high rate of misdiagnosis (7). They are often confused with ovarian and other pelvic tumors (8, 9), which can lead to misdiagnosis or missed diagnosis.

Myomectomy was the surgical procedure for removing uterine myomas from the abdominal cavity, typically performed after rotary cutting with a morcellator. In 2014, the U.S. Food and Drug Administration (FDA) issued a warning regarding the use of morcellation devices and advised caution when performing laparoscopic uterine or uterine myoma tissue fragmentation techniques. This patient presented with laparoscopic power morcellation (LPM)-related with omental involvement, which occurred after two laparoscopic myomectomy procedures at other hospital.

In recent years, with the widespread adoption of laparoscopic technology, laparoscopic myomectomy has become a common clinical treatment for uterine myoma. Compared to laparoscopic surgery, open surgery offers better tactile sensitivity to the lesion,which may help reduce the recurrence of uterine myoma. Moreover, although minimally invasive surgery allows for faster recovery, it is subject to strict limitations regarding the number, location, and size of myomas. The choice between laparoscopic and open surgery for treating uterine myoma depends on the surgeon's skills and experience, as well as the patient's individual condition. On the other hand, the outcome depends on doctor-patient communication, and patients should be fully informed before surgery that the use of a laparoscopic fibroid morcellator during the procedure may increase the risk of tumor cell dissemination. Doctors and patients need to collaborate in clinical decision-making, thoroughly evaluating the risks and benefits of different surgical methods to determine the best approach. Laparoscopic myomectomy offers advantages such as a wide and clear surgical field, minimal trauma, reduced bleeding, less pain, fewer adhesions, and faster recovery (10, 11), enabling patients with benign tumors to achieve minimally invasive surgical outcomes. However, after laparoscopic myomectomy, placing the fibroid in a closed retrieval bag for rotational resection still carries risks, including bag damage, leakage and splashing of the surgical fluids, and implantation at the abdominal incision site (12). Proper preoperative evaluation, selection of appropriate surgical methods, and strict adherence to tumor-free principles and techniques throughout the entire surgical process are essential for the effective treatment of myomectomy.

Fibroid morcellation refers to a surgical technique in which the uterus or leiomyomas are removed from the abdominal cavity via laparoscopy using a high-speed rotating electric morcellator to facilitate the extraction of “benign uterine tumors” (13–15). During this procedure, there is a risk of tumor tissue or cell implantation within the abdominal cavity, potentially leading to the spread of malignant tumors or recurrence of uterine myomas. In 1993, Steiner et al. (16) first reported the use of laparoscopic electric morcellators, noting that the device was safe and effective. In 1995, laparoscopic electric morcellators were approved by the FDA for clinical use. The fibroid morcellator addressed the challenge of time-consuming and complex removal of larger tumors through small abdominal incisions, thereby enhancing the benefits of laparoscopic surgery. However, it also increased the risk of tumor dissemination and implantation metastasis. In 2014, the US FDA issued a warning and completely halted the use of fibroid morcellators in laparoscopic myomectomy (17). In 2016, the New England Journal of Medicine published an article questioning whether the FDA's ban on electric morcellators might have been “overcorrected” (18). “Consensus on Laparoscopic Uterine (Tumor) Fragmentation” from the European Society of Gynecological Oncology (ESGO) in 2017, and “the updated safety notice on laparoscopic myomectomy” from the US FDA in 2020 recommended the use of closed containment bags to enable minimally invasive surgery while preventing tissue implantation and dissemination within the abdominal cavity (19). Studies had shown that placing fibroid specimens in homemade specimen bags for morcellation results in similar surgical and hospitalization times compared to traditional surgery (*p* > 0.05) (20). Histological examination of the junction between endometrial stromal or smooth muscle tumors and the surrounding muscle layer was crucial for classifying atypical stromal tumors. However, after uterine (fibroid) fragmentation surgery, it was often impossible to fully assess the size of the uterine tumor and the depth of epithelial or stromal lesions infiltrating the uterine muscle layer (21). This limitation increased the risk of incomplete or missed sampling, potentially leading to missed or misdiagnosed pathological results. Tumor-free techniques were essential to prevent the spread of cancer cells through the bloodstream, lymphatic system, and wound implantation. When using a fibroid morcellator, tumor-free techniques must be rigorously applied (22). In this study, surgical records from the other hospital indicated that a containment bag was used; nevertheless, leiomyomatosis peritonealis disseminata subsequently developed. We were considering whether this was related to incomplete containment or leakage. Before completing the surgery, a large volume of distilled water or physiological saline should be used to repeatedly and carefully rinse the pelvic and abdominal cavities to minimize the risk of implantation.

LPD was a non metastatic, homologous, multicenter benign tumor disease characterized by small smooth muscle tumors scattered on the surfaces of the peritoneum and omentum (23–25). Laparoscopic uterine fibroid fragmentation surgery had become an iatrogenic cause of LPD implantation. The incidence of LPD following laparoscopic uterine fibroid fragmentation surgery ranges from approximately 0.1% to 1.0% (26, 27). After myomectomy, small tissue fragments and fibroid cells remaining in the pelvic and abdominal cavities might be shed. Under various influences, these residual fibroid cells could proliferate, formed new blood vessels, and obtained blood supply from surrounding tissues, resulting in the formation of LPD nodules (28). These nodules were typically discovered only when corresponding symptoms and signs appeared. The incidence of LPD after unprotected uterine fibroid fragmentation surgery was approximately 0.12% to 0.95%, with an average diagnosis time of about 48 months or longer post-surgery (11). Li et al. (29) summarized 13 patients with LPD, among whom 11 had a history of laparoscopic uterine fibroid fragmentation surgery and were considered to have iatrogenic causes. Notably, 2 patients also had implanted LPD nodules near the laparoscopic trocar port in addition to intra-abdominal implantation nodules. Puthiyidom et al. (30) reported cases of LPD following hysteroscopic myomectomies performed with an intrauterine shaver and resectoscope. Although LPD was histologically benign, there was a risk of recurrence and malignant transformation. LPD might mimic malignancies such as leiomyosarcoma, and its nonspecific imaging and clinical features maked diagnosis challenging (31). LPD was a potentially malignant condition with a long latency period before transformation (32). The risk of malignant transformation in LPD ranges from 2% to 5%, with an interval of 1 month to 8 years between initial diagnosis and malignant transformation (33, 34). Rosati et al. (35) found that enlarged LPD nodules, with a median diameter of 12 cm should raise suspicion for malignancy. During the physical examination of this patient, ultrasound revealed a pelvic mass, with no gastrointestinal discomfort or anemia reported. Preoperative external MRI suggested a high likelihood of uterine fibroids. However, it was challenging to make a definitive diagnosis based solely on imaging without considering LPD prior to surgery. The patient in this case underwent two laparoscopic myomectomy in other hospital, with surgical records indicating the use of protective measures during both procedures. LPD with omental involvement developed 1 year after the second surgery. We were considering whether this was related to incomplete containment or leakage. The exact mechanism of dissemination cannot be definitively confirmed based on this single case alone. During the surgical procedure, all steps should be performed meticulously, the principle of tumor-free surgery should be strictly upheld, and the occurrence of LPD should be prevented.

## 4 Conclusion

In summary, even when using a containment bag, dissemination may still occur. And some patients may experience recurrence several years after surgery and require a second operation. Although this does not typically affect the patient's overall health, it can cause physical harm. If there is a high suspicion of sarcoma before surgery, open surgery can be preferred to reduce tumor dissemination caused by CO_2_ pneumoperitoneum. Surgical records from the referring hospital suggest that a containment bag was used; nevertheless, leiomyomatosis peritonealis disseminata subsequently developed. The exact mechanism of dissemination cannot be definitively confirmed by this single case alone. Additionally, close postoperative follow-up is essential to prevent recurrence and malignant transformation. As healthcare professionals, we should strive to minimize iatrogenic complications and adverse effects to ensure that patients truly benefit from minimally invasive surgery.

## Data Availability

The original contributions presented in the study are included in the article/Supplementary Material, further inquiries can be directed to the corresponding authors.
